# Quality of life in Brazilian children with food allergy

**DOI:** 10.1186/1939-4551-8-S1-A112

**Published:** 2015-04-08

**Authors:** Renata Guedes Castro Cunha, Wilson Rocha Filho, Analice Val Paula, Rosiléa Alves Silva

**Affiliations:** 1 Hospital Infantil João Paulo II – Fhemg, Brazil

## Background

The aim of his study is to test–retest the EuroPrevall quality of life questionnaire (QoLQ), and determine the influence of nutritional instructions, access to epinephrine, and the presence of atopic dermatitis on quality of life of patients with food allergy. Furthermore, we assessed whether patients with food allergy IgE-mediated and non-IgE mediated, presented difference in quality of life.

## Methods

This is a descriptive, cross-sectional study, where data collection was performed during 1 year. The questionnaire consists of questions classified among three domains: emotional impact, food anxiety, social and dietary restrictions. The average score of the three domains was obtained and the final quality of life score was calculated. Quality of life was classified into three categories, good, fair and poor. In order to assess the internal consistency of the QoLQ, its Cronbach's alpha coefficient was calculated. Pearson’s correlation coefficients of the total between 2 applications of the QoLQ were computed. Comparisons between and qualitative characteristics were performed using Fisher's Exact test.

## Results

Sixty children aged 0 to 14 years were analyzed, all treated as an outpatient in a food allergy clinic. Initially, 25 patients completed the questionnaire twice with an interval of 1 week. In both applications, the internal consistency was considered very good (coefficient greater than 0.9), which attests its reliability. Strong positive correlations between total QoLQ were obtained. Of the 60 patients, 22 (36.7%) of them were allocated within the group with good quality of life, 28 (46.7%) in the group of fair quality of life, and 10 (16.6%) were considered to have poor quality of life. There was no significant influence on the quality of life after nutritional instruction, adrenaline availability and atopic dermatitis comorbidity.

## Conclusions

The questionnaire used in this study was reliable for assessing individual patients and the comparison between studies. We have a large number of patients with quality of life rated as fair or poor. There was no association between quality of life and nutritional instruction, supply of epinephrine and the presence of atopic dermatitis.

